# Anti-inflammatory activities of *Ganoderma lucidum (Lingzhi)* and *San*-*Miao*-*San* supplements in MRL/lpr mice for the treatment of systemic lupus erythematosus

**DOI:** 10.1186/s13020-016-0093-x

**Published:** 2016-04-29

**Authors:** Zhe Cai, Chun Kwok Wong, Jie Dong, Delong Jiao, Man Chu, Ping Chung Leung, Clara Bik San Lau, Ching Po Lau, Lai Shan Tam, Christopher Wai Kei Lam

**Affiliations:** Department of Chemical Pathology, The Chinese University of Hong Kong, Prince of Wales Hospital, Shatin, N.T., Hong Kong, China; Shenzhen Research Institute, The Chinese University of Hong Kong, Shenzhen, China; Institute of Chinese Medicine and State Key Laboratory of Phytochemistry and Plant Resources in West China, The Chinese University of Hong Kong, Hong Kong, China; Department of Medicine and Therapeutics, Prince of Wales Hospital, The Chinese University of Hong Kong, Shatin, Hong Kong, China; State Key Laboratory of Quality Research in Chinese Medicine, Macau Institute for Applied Research in Medicine and Health, Macau University of Science and Technology, Taipa, Macau, China

## Abstract

**Background:**

*Ganoderma lucidum* (*Lingzhi*; LZ) and *San*-*Miao*-*San* (SMS) are Chinese medicines (CMs) used to treat inflammatory ailments and numbing syndrome/arthralgia syndrome (*Bi Zheng*), respectively. Given that the main symptoms of systemic lupus erythematosus (SLE) include inflammation of the joints, joint pain, edema and palpitations of the heart because of problems associated with *Bi Zheng*, it was envisaged that LZ and SMS could be used as potential treatments for this autoimmune disease. This study aims to investigate the anti-inflammatory activity of a combination formulation containing LZ and SMS (LZ–SMS) in SLE mice.

**Methods:**

Female adult Balb/c mice of 20–24 weeks of age were used as normal mice (n = 10), whereas female MRL/lpr mice of 12–24 weeks of age were divided into three groups (n = 10 in each group), including mild, moderate and severe SLE mice groups. The clinical characteristics of the SLE and Babl/c mice (i.e., body weight, joint thickness, lupus flare, proteinuria, leukocyturia and lymphadenopathy) were assessed. The plasma concentrations of anti-nuclear antibody (ANA) and anti-double stranded DNA antibody (anti-ds-DNA) were analyzed by an enzyme-linked immunosorbent assay, whereas the concentration of several key cytokines (IFN-γ, TNF-α, IL-6, IL-10, IL-2, IL-27, IL-12P70, IL-17A and IL-21) were analyzed by a Luminex multiplex assay. The gene expression profiles for differentiation of the T helper (Th) lymphocytes in splenic CD4^+^ Th cells were assessed by RT-qPCR. Flow cytometry was used to measure the percentages of CD4^+^CD25^+^Foxp3^+^ Treg cells and CD19^+^CD5^+^CD1d^+^IL-10^+^ regulatory B (Breg) cells (IL-10^+^ Bregs).

**Results:**

Concentrations of anti-ds-DNA in the plasma samples collected from the LZ–SMS-treated (500 mg/kg/day oral administration for 7 days followed with 50 mg/kg/day intraperitoneal administration for 7 days), moderate and severe SLE mice decreased significantly compared with the PBS treated mice (*P* < 0.05). The gene expression levels of the induced regulatory T (iTreg) and natural Treg (nTreg) cells were significantly higher than those of the Th17, Th1 and “conventional Th cells vs. Treg cells” regulated genes following the LZ–SMS treatment (*P* < 0.05). The percentages of CD4^+^CD25^+^Foxp3^+^ Treg cells collected from the splenic, thymic and peripheral blood cells, as well as the percentages of IL-10^+^ Bregs collected from the splenic and thymic cells increased significantly in the LZ–SMS-treated SLE mice (*P* < 0.05) compared with the untreated PBS group. The ratio of the percentage of CD4^+^CD25^+^Foxp3^+^ Treg cells to the percentage of CD4^+^CD25^–^ effector T cells collected from the splenic, thymic and peripheral blood cells in LZ–SMS-treated moderate and severe SLE mice increased significantly compared with the untreated PBS group (*P* < 0.05). Furthermore, a comparison with the PBS treatment group revealed significant decreases in the concentrations of several inflammatory cytokines, including IL-21, IL-10 and IL-17A (*P* < 0.05), as well as significant increases in the concentrations of IL-2 and IL-12P70 in the LZ–SMS treated SLE mice (*P* < 0.05).

**Conclusion:**

LZ–SMS treatment led to significant increases in the percentages of CD4^+^CD25^+^Foxp3^+^ Treg and IL-10^+^ Breg cells, together with a reduction in the plasma concentrations of several inflammatory cytokines and the down-regulated expression of the corresponding cytokine related genes in SLE mice. The clinical characteristics of the LZ–SMS-treated SLE mice also improved significantly.

**Electronic supplementary material:**

The online version of this article (doi:10.1186/s13020-016-0093-x) contains supplementary material, which is available to authorized users.

## Background

Systemic lupus erythematosus (SLE) is a multi-systemic chronic autoimmune relapsing disease with a particularly high prevalence in middle-aged women, where it often results in damage to multiple organs, including the kidneys, skin, heart, blood vessels and central nervous system [1]. Although several drugs have been developed for this disease, which have led to considerable improvements in overall survival as well as alleviating the symptoms of severe organ damage, they are expensive and produce unwanted side effects [2–5].

*Ganoderma lucidum* (*Lingzhi*; LZ) has been reported to exhibit antioxidant, anti-inflammatory and analgesic effects with potential therapeutic benefits against a wide range of diseases, including hepatitis, hypertension, arthritis, bronchitis and malignancy [6–13]. LZ induced the expansion of both murine and human CD4^+^ T cells into Foxp3^+^ regulatory T (Treg) cells, which could be used to alleviate acute colitis [14]. LZ has also been reported to modulate the activity of peripheral mononuclear cells and suppress the production of SLE-related pathogenic cytokines, such as tumor necrosis factor-α (TNF-α), as well as interleukin (IL)-1β, IL-12 and IL-6 [5, 15–17].

*San*-*Miao*-*San* (SMS) is a Chinese medicine (CM) containing a 1:1:1 (w:w:w) mixture of three Chinese herbs, including *Rhizoma atractylodis* (*Cangzhu*), *Cortex phellodendri* (*Huangbai*) and *Radix achyranthis bidentatae* (*Niuxi*) [18]. This material has been used empirically for treating numbing syndrome/arthralgia syndrome (*Bi Zheng*), which is a painful obstructive syndrome caused by the invasion of exterior pathogenic factors into the muscles, tendons, bones and joints of the sufferer [5, 18]. According to CM, SLE can be generally classified into excessive and deficient syndromes and related to *Bi Zheng*, because this syndrome results in joint problems, joint pain, edema and palpitations of the heart because of its damaging effects [19]. It was therefore envisaged that the combination of SMS with *Lingzhi* could be beneficial for the treatment of SLE. The combined use of LZ and SMS (LZ–SMS) for their anti-inflammatory activities was once common practice according to CM herbalists because this combination of materials exhibits synergistic therapeutic effects [20]. Furthermore, the results of several clinical studies showed that a combined standard formulation of LZ and SMS was generally safe and well tolerated by patients suffering from rheumatoid arthritis (RA) [18, 21]. However, the in vivo anti-inflammatory mechanisms of LZ and SMS for the treatment of SLE have not yet been investigated.

We previously investigated the intracellular signal transduction mechanisms responsible for regulating the activation of human eosinophils, epithelial cells and fibroblast-like synoviocytes (FLS) during inflammation, and studied the pathological roles of cytokines in allergic and autoimmune diseases [22–25]. This study aims to investigate the anti-inflammatory activities of this combined formulation of LZ–SMS in SLE mice.

## Methods

### Materials

The combined LZ–SMS hot water extract formulation used in this study contained a lyophilized powder, which was prepared according to a previously reported procedure by researchers at the Institute of Chinese Medicine, The Chinese University of Hong Kong, China [18]. This extract was prepared by combining LZ 35.7 % with SMS, which consists of three Chinese herbs, including *Cangzhu* 21.4 %, *Huangbai* 21.4 % and *Niuxi* 21.4 %. The preparation was then dissolved in physiological saline, and the resulting solution was analyzed by a Limulus amoebocyte lysate assay (sensitivity limit 12 pg/mL; Biowhittaker Inc, Walkersville, MD, USA), which revealed that it did not contain any detectable lipopolysaccharide. The different herbs were authenticated in terms of their chemical composition by thin layer chromatography against a reference herb and several reference chemical markers, according to the Chinese Pharmacopoeia 2010 [26].

### Mice

Female MRL/MpJ-Fas^lpr^/2 J (MRL/lpr) mice were purchased from the Jackson Laboratory (Bar Harbor, ME, USA) and maintained under specific pathogen-free conditions in the Laboratory Animal Services Center (LASC), The Chinese University of Hong Kong (CUHK) and Cancer Center of Prince of Wales Hospital, Hong Kong. Female adult Balb/c mice (LASC, CUHK) of 20–24 weeks of age were used as normal mice (n = 10). Adult female MRL/lpr SLE mice of 12–24 weeks of age (n = 30) were kept in a conventional animal facility. All of the experiments conducted in the present study involving live animals were carried out under strict controls according to the Animal Experimentation Ethics Committee Guide for the Care and Use of Laboratory Animals, as approved by the Animal Experimentation Ethics Committee of CUHK (Ethical approval no.: 14-012-MIS) (Additional file 1).

### Monitoring of disease activity

The urine samples collected from the different groups (n = 10) of mice were analyzed for protein and leukocytes by URS-5T urine test strips (Healgen Scientific LLC, Houston, TX, USA). Female MRL/lpr mice of 12–24 weeks of age were divided into three groups (n = 10 in each group), including mild, moderate and severe SLE mice according to the following proteinuria scoring system: score 0 0–15 mg/dL, score 1 16–29 mg/dL, score 2 30–99 mg/dL, score 3 100–299 mg/dL, score 4 300–1999 mg/dL, and score 5 ≥2000 mg/dL (score 0–1: mild SLE; score 2–3: moderate SLE and score 4–5: severe SLE) [27].

### Biochemical and physiological parameters

The different groups of MRL/lpr and Balb/c mice (both groups n = 5) were used to investigate the nephritic effects of the LZ–SMS formulation on the mice following the daily oral and intraperitoneal administration of this material for 14 days. The oral administration group was given 500 mg/kg/day of the LZ–SMS formulation in 400 μL of phosphate buffered saline (PBS) from day 1 to 7 (n = 5 for the lupus mice in each SLE group and Balb/c mice in the normal group). A 50 mg/kg/day dose of LZ–SMS was also prepared in 400 μL of PBS because of its limited solubility. This dose was intraperitoneally administered from day 8 to 14 into the lupus mice or normal mice (n = 5 in each case) belonging to each group. For the intraperitoneal injection of this lower dose of LZ–SMS, we only injected clear solutions of the LZ–SMS formulation (about 1.25 mg LZ–SMS/mouse daily), which were filtered prior to being injected to remove any insoluble materials. PBS was given to each mouse on a daily basis at a dose of 400 μL/mouse for 14 days according to the same protocol as that used for the PBS control (n = 5 of each group). The body weight of each mouse was measured before and after the administration of the LZ–SMS formulation to provide some indication of possible herbal toxicity [28]. Mice were also culled 1 day after the administration of the LZ–SMS formulation to investigate several immunological parameters, including the plasma concentrations of anti-nuclear antibody (ANA), anti-double stranded DNA antibody (anti-ds-DNA) and several cytokines (IL-2, IL-10, IL-27, IL-21, IL-12P70, IFN-γ, TNF-α, IL-17A and IL-6) in the whole blood. Clinical signs of lupus flare and disease severity were assessed in each group (n = 10) of MRL/lpr mice before and after the treatment process using the following four-point scale: 0–3, where 0 is normal, 1 is mild (affected areas including the snout and ears), 2 is moderate (affected area <2 cm on the snout, ears and intrascapular region) and 3 is severe (affected area >2 cm on the snout, ears and intrascapular region) [29].

### Histopathological assessment

Longitudinal sections of the kidneys, which were cut through the papilla, were fixed in 4 % paraformaldehyde buffer and embedded in paraffin. The kidney sections were subsequently cut into 2-μm-thick slices and stained with periodic acid-Schiff (PAS) stain. The number of mesangial cells in the glomeruli was determined by counting their nuclei. Microscopy examination (Leica Microsystems, Wetzlar, Germany) was used to evaluate the area of the glomeruli, with 100 glomeruli being evaluated in each group. The severities of vessel infiltration, interstitial nephritis and glomerulonephritis were scored using the following macroscopic scoring system: 0, normal; 1–2, mild; 3, moderate; and 4–5, severe [30].

### Quantitative real-time polymerase chain reaction (RT-qPCR)

Whole transcriptome analysis for the differentiation of the T helper (Th) cells was performed using a mouse T helper cell differentiation RT^2^ profiler PCR array (Qiagen GmbH, Hilden, Germany). The RNA was extracted from splenic CD4^+^ Th cells by an RNeasy Mini Kit (Qiagen). The splenic CD4^+^ Th cells were purified by a mouse T helper cell isolation kit (Miltenyi Biotec, Bergisch Gladbach, Germany) with Th cell purity >95 %. The purified RNA from 1 × 10^6^ splenic CD4^+^ Th cells were analyzed according to the manufacturer’s instructions for the RT^2^ profiler PCR Array kit by a 384-well Applied Biosystems 7900HT Fast Real-Time PCR System (Applied Biosystems Inc., Foster City, CA, USA). The mRNA expression levels were calculated as follows:

$$\frac{{\frac{{2^{{{\text{ }} - {\text{ }}\Delta {\text{C}}_{{\text{T}}} ({\text{GOI}})}} {\text{expt}}}}{{2^{{{\text{ }} - {\text{ }}\Delta {\text{C}}_{{\text{T}}} ({\text{HKG}})}} {\text{expt}}}}}}{{\frac{{2^{{{\text{ }} - {\text{ }}\Delta {\text{C}}_{{\text{T}}} ({\text{GOI}})}} {\text{ctrl}}}}{{2^{{{\text{ }} - {\text{ }}\Delta {\text{C}}_{{\text{T}}} ({\text{HKG}})}} {\text{ctrl}}}}}} = \frac{{2^{{ - [{\text{C}}_{{\text{T}}} ({\text{GOI}}){\text{ }} - {\text{ }}\Delta {\text{C}}_{{\text{T}}} ({\text{HKG}})]}} {\text{expt}}}}{{2^{{ - [{\text{C}}_{{\text{T}}} ({\text{GOI}}){\text{ }} - {\text{ }}\Delta {\text{C}}_{{\text{T}}} ({\text{HKG}})]}} {\text{ctrl}}}} = \frac{{2^{{{\text{ }} - {\text{ }}\Delta {\text{C}}_{{{\text{T}}_{{{\text{expt}}}} }} }} }}{{2^{{{\text{ }} - {\text{ }}\Delta {\text{C}}_{{{\text{T}}_{{{\text{ctrl}}}} }} }} }} = 2^{{{\text{ }} - {\text{ }}\Delta \Delta {\text{C}}_{{\text{T}}} }}$$

where expt is the experiment, ctrl is the control, GOI is the gene of interest and HKG is the housekeeping gene.

### Flow cytometric analyses of the CD4^+^CD25^+^Foxp^+^ Treg cells, CD4^+^CD25^−^ effector T (Teff) cells and CD19^+^CD5^+^CD1d^+^IL-10^+^ Breg cells

Peripheral blood, splenic and thymic cells (1 × 10^6^ cells in each case) from the MRL/lpr and Balb/c mice were stained to determine the number of CD4^+^CD25^+^Foxp^+^ Treg cells [fluorescein peridinin chlorophyll protein/PerCP-conjugated CD4^+^, allophycocyanin/APC-conjugated CD25^+^ (BioLegend, San Diego, CA, USA) and Alexa Fluor 488-conjugated Foxp^+^ (BD Pharmingen Corp., San Diego, CA, USA) T lymphocyte sub-populations], CD4^+^CD25^–^ Teff cells and IL-10^+^ Breg cells [(CD19^+^CD5^+^CD1d^+^ regulatory B cell) cocktail and Phycoerythrin/Cyanine Dye 7 (PE/Cy7)-conjugated anti-IL-10 antibody (BioLegend)] by flow cytometry (Beckman Navios flow cytometer, Beckman Coulter Inc., Brea, CA, USA).

### Plasma concentrations of ANA and anti-ds-DNA

The plasma ANA and anti-ds-DNA concentrations in each group were measured by an enzyme-linked immunosorbent assay (ELISA) with commercial reagent kits (Mybiosource, Inc., San Diego, CA, USA).

### Plasma cytokine concentrations of MRL/lpr and Balb/c mice

Plasma was harvested and stored at –80 °C prior to being subjected to a multiplex immunoassay to measure several cytokines (i.e., IL-2, IL-10, IL-27, IL-21, IL-12P70, IFN-γ, TNF-α, IL-17A and IL-6) using a Milliplex MAP kit assay reagent (Merck Millipore, Billerica, MA, USA) with a Bio-Plex 200 suspension array system (Bio-Rad Laboratories, Hercules, CA, USA).

### Statistical analysis

The results for the statistical analysis of the gene array data are described in the gene array hybridization and data analysis section. These results have been expressed as the mean ± standard deviation (SD) for normally distributed data. Non-parametric data have been expressed as the median values (interquartile range, IQR). Mann–Whitney U tests were used for the continuous variables. A comparison of the different groups was achieved by Kruskal–Wallis ANOVA, followed by Dunn’s post-test for comparing the differences and calculating a *P* value for each pair of comparison. Two-way ANOVA was used to compare the different groups and treatments, followed by Bonferroni post-test analysis for comparing the replicate means and calculating a *P* value for each data pair. All hypotheses were two-tailed, and *P* values less than 0.05 were considered significant. All of these data were analyzed by the 6.0 for Windows version of the GraphPad Prism software (GraphPad Software, La Jolla, CA, USA).

## Results

### SLE disease characteristics of MRL/lpr mice

The clinical characteristics of the different mice are summarized in Table 1. The MRL/lpr mice treated with PBS were initially stratified into three subgroups (i.e., mild, moderate or severe) based on their disease activity, which provided a reflection of their proteinuria scores. The percentages of lymphadenopathy and the body weights in the different groups of MRL/lpr mice were significantly higher than those of the control mice (*P* = 0.034). Furthermore, no obvious adverse side effects were observed in the MRL/lpr mice used in the present study, as evidenced by the lack of significant loss of body weight upon LZ–SMS administration, all *P* > 0.05. All of the MRL/lpr mice in the moderate (*P* < 0.001) and severe (*P* = 0.035) groups had significantly higher proteinuria and leukocyturia scores than the control mice. However, there were no significant differences in these scores between the mild and control groups (*P* = 0.056). The joint thickness and lupus flare scores of the MRL/lpr mice in the moderate (*P* = 0.036) and severe (*P* = 0.030) groups were significantly higher than those of the control group.

**Table 1:** Clinical characteristics of MRL/lpr and Balb/c mice before treatment

	MRL/lpr (n = 30)		Balb/c	
	Mild (n = 10)	Moderate (n = 10)	Severe (n = 10)	Normal (n = 10)
*Clinical assessment*				
Body weight, mean ± SD, g	48.0 ± 8.7^&^	49.4 ± 9.1**	48.7 ± 4.1^#^	19.2 ± 0.2
Joint thickness, mean ± SD, μm	84.3 ± 3.1	84.7 ± 2.7*	84.4 ± 2.4	79.3 ± 3.6
Lupus flare score, mean ± SD	0.7 ± 0.5	1.0 ± 0.9	1.3 ± 1.0^#^	0
Proteinuria score, mean ± SD	1.5 ± 0.5	2.8 ± 0.4***	4.0 ± 1.1^###^	0.7 ± 0.6
Leukocyturia score, mean ± SD	0.5 ± 1.2	2.0 ± 1.4*	2.5 ± 0.5^##^	0
Lymphadenopathy, No. (%)	10/10 (100 %)^&&&^	10/10 (100 %)***	10/10 (100 %)^###^	0/10 (0 %)

Joint thickness was measured in three joints of each hind paw (tarsus joint); Leukocyturia score was determined by a four-point scale (0–3, where 0 was normal and 3 was severe); No. (%), percentages of mice had lymphadenopathy among each group

*SD* standard deviation, *MRL/lpr* MRL/MpJ-Fas^lpr^/2 J, *Mild* mice with mild disease activity (mild SLE, proteinuria score is 0–1), *Moderate* moderate disease activity (moderate SLE, proteinuria score is 2–3), *Severe* high disease activity (severe SLE, proteinuria score is 4–5), *Normal* age- and sex- matched healthy Balb/c mice

^&^ *P* = 0.03, ^&&^ *P* = 0.008, ^&&&^ *P* = 0.0008, comparing between control and mild SLE mice

* *P* = 0.03, ** P = 0.008, *** P = 0.0008, comparing between control and moderate SLE mice

^#^ *P* = 0.03, ^##^ *P* = 0.008, ^###^ *P* = 0.0008, comparing between control and severe SLE mice

### Attenuation of nephritis in MRL/lpr mice treated with LZ–SMS

**Fig. 1:**
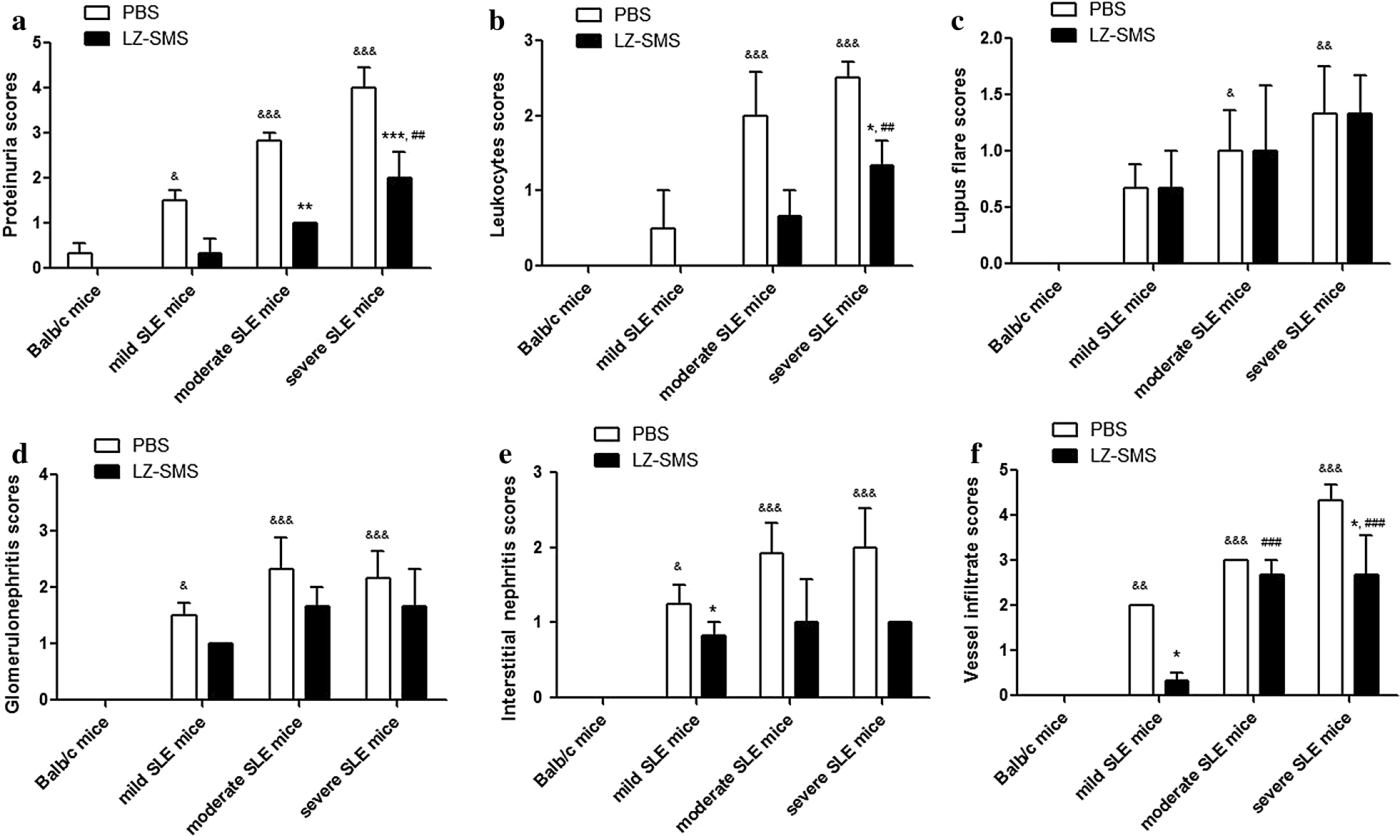
Clinical characteristics of MRL/lpr mice with LZ–SMS or PBS treatment. The results of each score system of signs **a** proteinuria, **b** leukocyturia, **c** lupus flare, **d** glomerulonephritis, **e** interstitial nephritis and **f** vessels infiltration are presented as bar charts showing the arithmetic mean plus SD. * <0.05, **P < 0.01, ***P < 0.001, PBS- vs. LZ–SMS-treated MRL/lpr mice; ^&^ *P* < 0.05, ^&&^ *P* < 0.01, ^&&&^ *P* < 0.001, Balb/c control mice vs. PBS-treated MRL/lpr mice; ^##^ *P* < 0.01, ^###^ *P* < 0.001, Balb/c control mice vs. LZ–SMS-treated MRL/lpr mice (n = 5 in each group)

**Fig. 2:**
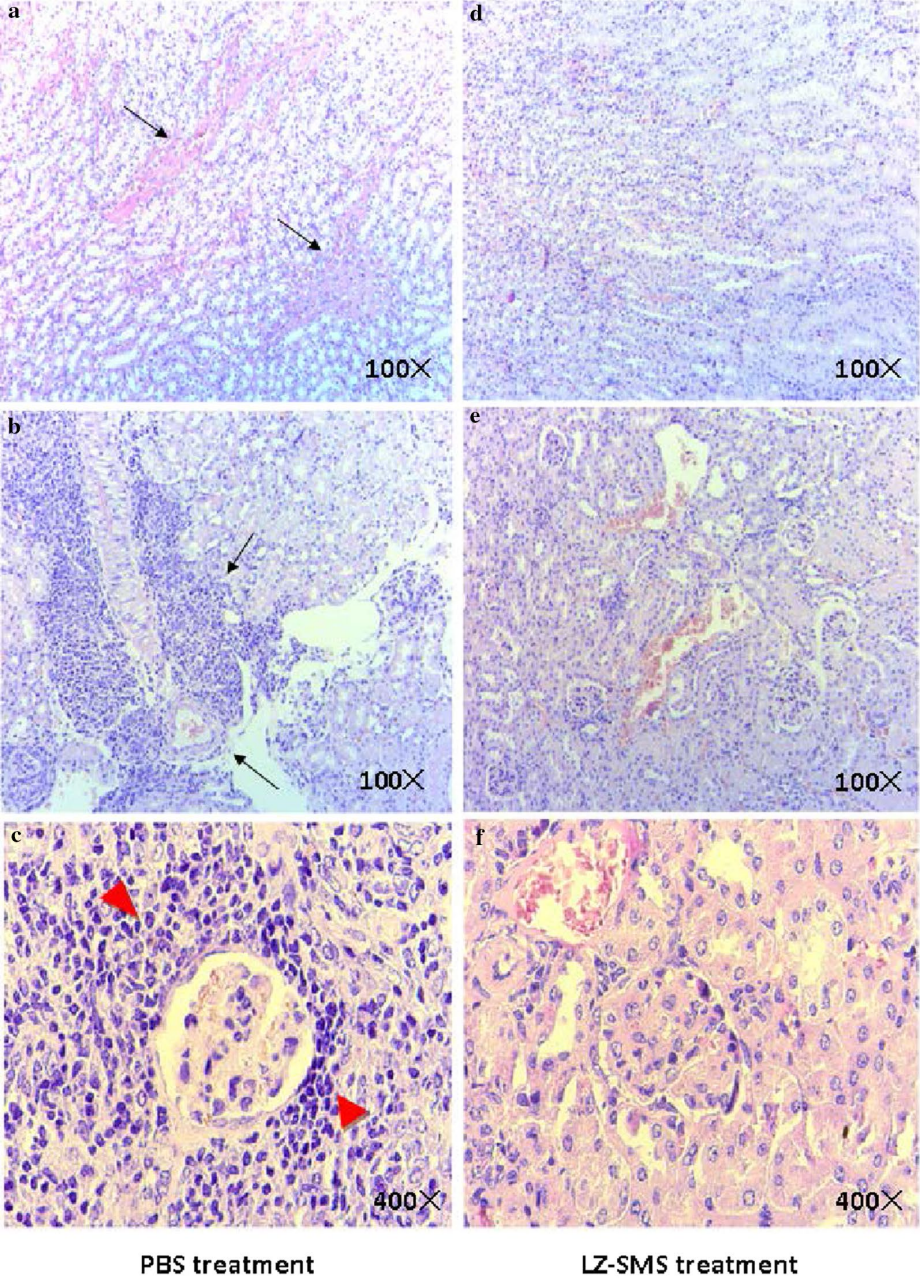
Effects of LZ–SMS treatment on nephritis disease in severe MRL/lpr mice. Kidney tissues from MRL/lpr mice were collected and fixed in 4 % paraformaldehyde. The tissue sections were stained with PAS. Representative kidney histopathology in (**a**–**c**) PBS and (**d**–**f**) LZ–SMS treated SLE mice are shown. **a**, **d** PAS staining of a representative renal tubular area is shown (100×). *Black arrow* depicts the protein cast in tubular area. **b**, **e** PAS staining of a representative perivascular area is shown (100×). *Black arrow* depicts the mononuclear cells infiltration on vessel. **c**, **f** PAS staining of a representative glomerulus area is shown (400×). *Bold red arrow* depicts the mononuclear cells infiltration round glomerulus (n = 5 in each group)

**Fig. 3:**
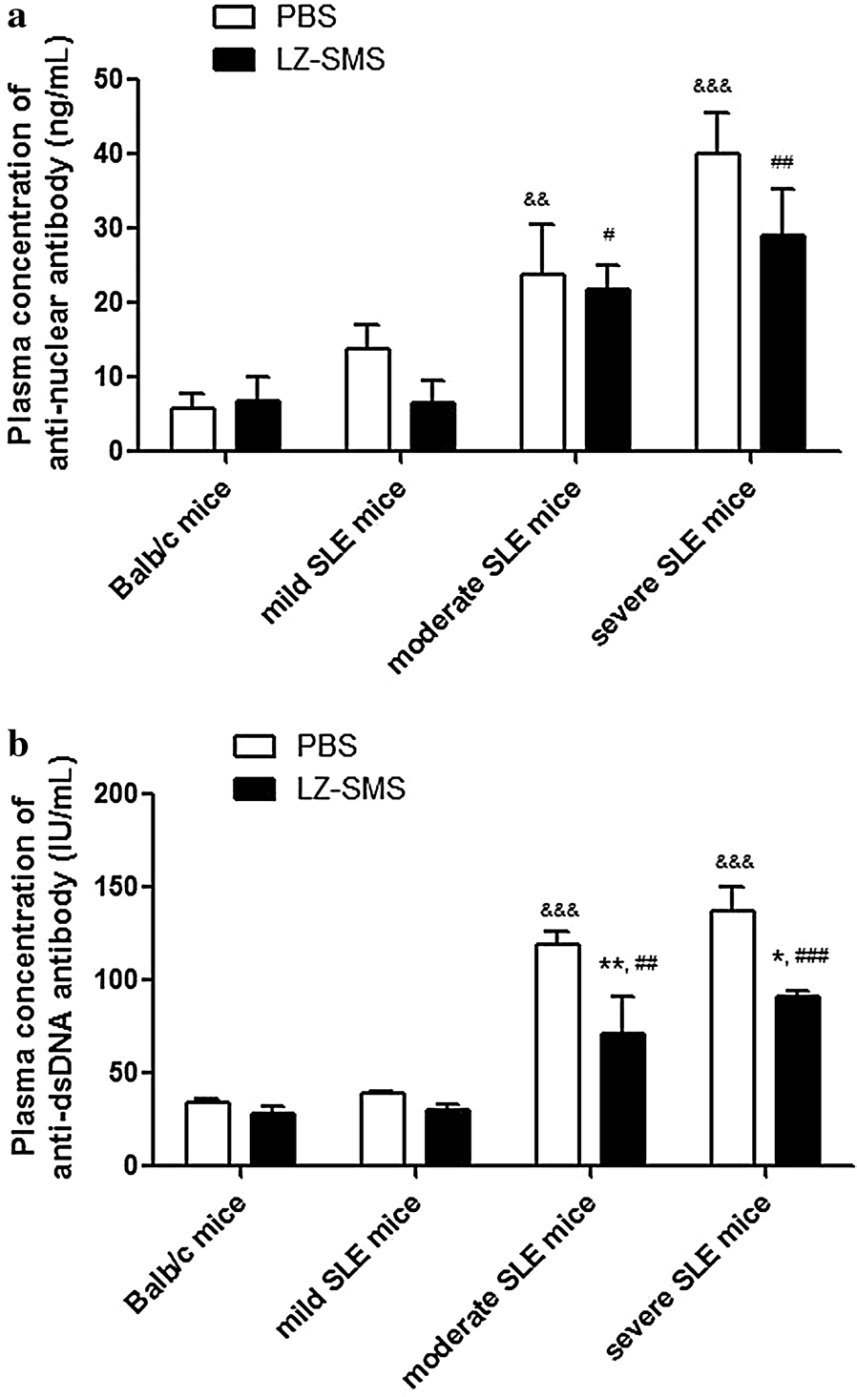
Plasma concentrations of ANA and anti-ds-DNA antibody in LZ–SMS or PBS-treated MRL/lpr and Balb/c mice. Concentrations of plasma **a** ANA and **b** anti-ds-DNA are presented as *bar charts* showing the arithmetic mean plus SD. ^*^ *P* < 0.05, ^**^ *P* < 0.01, PBS- vs. LZ–SMS-treated MRL/lpr mice; ^&&^ *P* < 0.01, ^&&&^ *P* < 0.001, Balb/c control mice vs. PBS treated MRL/lpr mice; ^#^ *P* < 0.05, ^##^ *P* < 0.01, ^###^ *P* < 0.001, Balb/c control mice vs. LZ–SMS treated MRL/lpr mice (n = 5 in each group)

To confirm the immunoregulatory and therapeutic roles of LZ–SMS in vivo, we measured the nephritis clinical characteristics of the MRL/lpr mice after they had been treated with LZ–SMS or PBS. As shown in Fig. 1, the proteinuria, leukocyturia, lupus flare, glomerulonephritis, interstitial nephritis and vessel infiltrate scores for the MRL/lpr mice without LZ–SMS treatment were significantly higher than those of the Balb/c control (all *P* < 0.05). Furthermore, the results for the LZ–SMS-treated MRL/lpr mice revealed that there were obvious remissions in the extent of the nephritic disease compared with PBS treatment alone, especially in the severe SLE mice (Figs. 1, 2). Moreover, there were significant differences in the plasma concentrations of ANA and anti-ds-DNA antibodies in the moderate and severe SLE mice compared with the Balb/c control mice treated with PBS (all *P* < 0.01) (Fig. 3). Although the plasma concentrations of ANA and anti-ds-DNA antibodies in the moderate and severe SLE mice treated with LZ–SMS were significantly higher than those of the Balb/c control mice, the plasma concentrations of anti-ds-DNA in the moderate (*P* < 0.001) and severe SLE mice (*P* = 0.05) were both significantly lower than those of the PBS-treated mice.

### Gene expression profile of Th cell differentiation

**Fig. 4:**
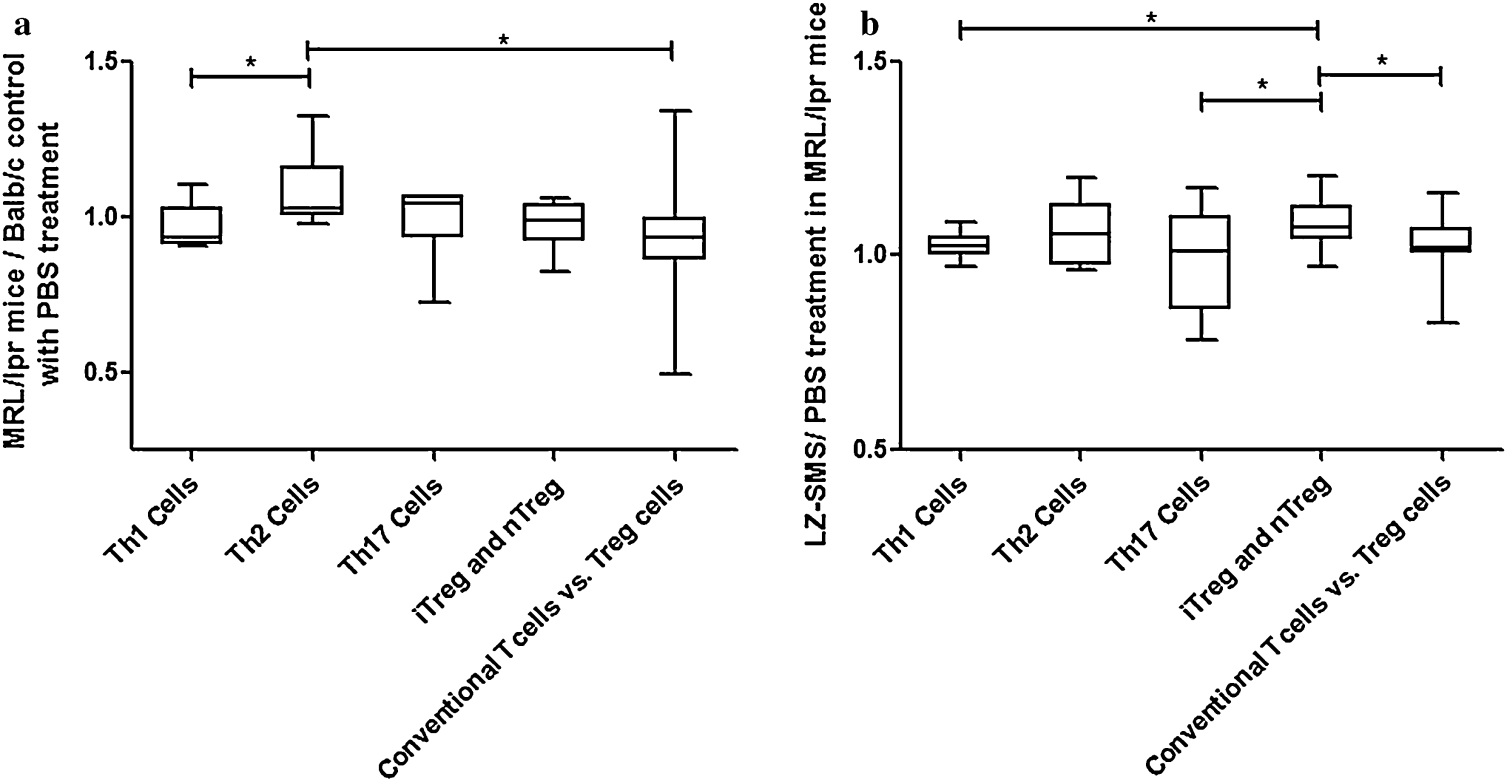
RT^2^ profiler PCR array of mouse Th cell differentiation. The results of gene expressions of Th1 cells, Th2 cells, iTreg and nTreg, Th17 cells and “conventional Th vs. Treg cells” in **a** PBS-treated MRL/lpr vs. Balb/c mice (in ratio) and **b** LZ–SMS-treated vs. PBS-treated MRL/lpr mice (in ratio) are presented as *box*-and-*whisker plots* with the median (IQR). Mann–Whitney U test was used to assess the differences of mRNA expression among different genes of Th cells. ^*^ *P* < 0.05

A common logarithm of gene expression revealed that the expression levels of Th1 cell-regulated genes (*Ifng, Il12rb2, Il18r1, Il18rap, Fasl* and *Tbx21*) and conventional Th cell *vs.* Treg cell-regulated genes (*Cacna1f, Chd7, Foxp3, Gata4, Hopx, Hoxa10, Hoxa3, Id2, Ikzf2, Il1r2, Il2ra, Lrrc32, Perp, Pkd2, Tnfrsf9, Trp53inp1, Uts2* and *Zeb1*) were significantly lower than those of the Th2 cell-regulated genes (*Asb2, Gata3, Il13, Il1rl1, Il4, Il5* and *Pparg*) in the splenic CD4^+^ Th lymphocytes from the PBS-treated MRL/lpr mice compared with the PBS-treated Balb/c control mice (Fig. 4a). However, following LZ–SMS treatment, the mean values of the induced regulatory (iTreg) and natural Treg (nTreg) (*Ccr6, Fosl1, Foxp3, Ikzf2, Il9, Irf4, Irf8, Myb, Nr4a1, Nr4a3, Pou2f2, Rel, Tgif1* and *Tnfsf11*) regulated gene expression levels in the splenic CD4^+^ Th lymphocytes obtained from MRL/lpr mice were significantly higher than those of the Th17 cell-regulated genes (*Il17a, Il17re, Il1r1, Il21, Rora* and *Rorc*) in Th1 cells compared with the PBS treatment group (Fig. 4b). These results implied that LZ–SMS was exhibiting an immunoregulatory role towards the differentiation of splenic CD4^+^ Th cells by inducing the differentiation of CD4^+^ Th cells into iTreg cells, whilst suppressing the differentiation of Th1 and Th17 cells in vivo. Most of the Th cell differentiation-related transcription factors, as well as the cytokine and receptor genes, were found to be up-regulated (ratio > 1.0) in the MRL/lpr mice belonging to the LZ–SMS treatment group compared with those in the PBS treatment group. This was especially true for the Treg differentiation-related genes IL-2 and Foxp3, which were up-regulated by more than 1.5-fold (Table 2).

**Table 2:** Th differentiation PCR array analysis

Transcription Factors		Cytokines and receptors	
Gene	LZ–SMS PBS	Gene	LZ–SMS PBS
Fosl1	1.94	Tnf	0.98
Nr4a1	5.12	Il1r2	1.94
Nr4a3	6.46	Il17re	1.10
Hoxa10	1.06	Il21	2.01
Rel	3.22	Ccr6	1.11
Irf4	2.03	Csf2	0.79
Nfatc1	1.88	Il4	1.12
Irf8	1.57	Il18r1	1.78
Id2	1.74	Ccr3	3.61
Gata4	1.99	Ccl5	0.96
Rora	5.04	Il13ra1	0.69
Cebpb	1.72	Il1rl1	0.82
Stat1	1.58	Il18	0.84
Zbtb7b	0.83	Il2ra	0.35
Pou2f2	1.40	Ccl7	0.73
Maf	2.14	Il18rap	2.27
Stat6	0.97	Ccr4	1.34
Rorc	1.19	Il12p40	0.53
Runx1	1.29	Il1r1	3.90
Gata3	1.68	Il2	2.63
Zeb1	1.03	Ccl11	1.38
Nfatc2	2.01	Il12rb2	2.68
Foxp3	1.91	Il4ra	1.99
Nfatc2ip	1.53	Il9	6.13
Runx3	2.00	Il13	1.99
Hoxa3	1.22		
Stat4	1.41		

cDNA samples were obtained from splenic cells of 24 weeks old female MRL/lpr mice 1 day after treatment. Values represent fold increase of LZ–SMS treatment (500 mg/kg/day of LZ–SMS formulation in 400 μL PBS for day 1–7 by oral administration followed by 50 mg/kg/day of soluble LZ–SMS in 400 μL PBS for day 8–14 by intraperitoneal route) versus the PBS treatment group

### Increase in the percentages of CD4^+^CD25^+^Foxp3^+^ Treg cells and IL-10^+^ Breg cells in MRL/lpr mice following LZ–SMS treatment

**Fig. 5:**
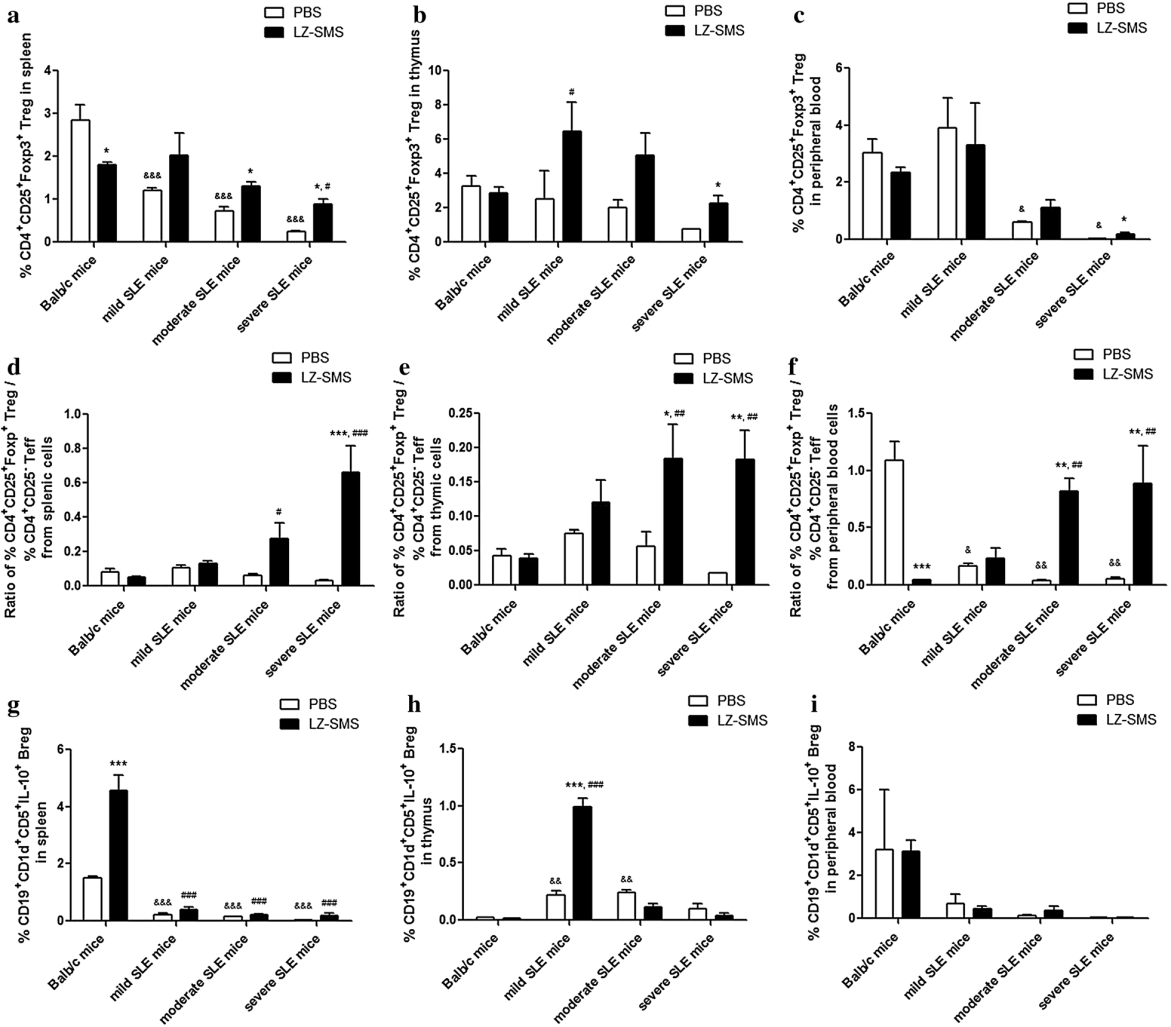
Characterization of CD4^+^CD25^+^Foxp3^+^ Tregs and IL-10 + Bregs in LZ–SMS or PBS-treated MRL/lpr and Balb/c mice. *Bar charts* show the arithmetic mean plus SD of the percentages of CD4^+^CD25^+^Foxp3^+^ Tregs in **a** splenic, **b** thymic and **c** peripheral blood cells; and the ratios of CD4^+^CD25^+^Foxp3^+^ Treg %/CD4^+^CD25^−^ effector T cell % in **d** splenic, **e** thymic and **f** peripheral blood cells; and the percentages of IL-10 + Breg cells in **g** splenic, **h** thymic and **i** peripheral blood cells. *P < 0.05, **P < 0.01, ***P < 0.001, LZ–SMS vs. PBS treatment; ^&^ *P* < 0.05, ^&&^ *P* < 0.01, ^&&&^ *P* < 0.001, Balb/c control mice vs. PBS-treated MRL/lpr mice; ^#^ *P* < 0.05, ^##^ *P* < 0.01, ^###^ *P* < 0.001, Balb/c control mice vs. LZ–SMS-treated MRL/lpr mice (n = 5 in each group)

We found that the number of CD4^+^CD25^+^Foxp3^+^ Treg cells in the different groups of MRL/lpr mice decreased in a linear manner compared with the Balb/c control mice both with and without LZ–SMS treatment (Fig. 5a–c). Compared with the PBS treatment group, the percentages of CD4^+^CD25^+^Foxp3^+^ Treg cells obtained from the splenic, thymic and peripheral blood cells in the severe SLE mice treated with LZ–SMS increased significantly (all *P* < 0.05). Furthermore, the percentage of CD4^+^CD25^+^Foxp3^+^ Treg cells obtained from the splenic cells in the moderate SLE mice treated with LZ–SMS was significantly higher than the value achieved with PBS treatment (*P* = 0.004). Accordingly, the percentage ratios of the CD4^+^CD25^+^Foxp3^+^ Treg cells to the CD4^+^CD25^–^ effector T cells obtained from the splenic, thymic and peripheral blood cells in the severe SLE mice treated with LZ–SMS, and from thymic and peripheral blood cells in moderate SLE mice with LZ–SMS treatment were significantly increased compared with PBS treatment group (Fig. 5d, e). Finally, there was a significant increase in the percentage of IL-10^+^ Breg cells obtained from the thymic cells in mild SLE mice treated with LZ–SMS compared with the PBS-treated mice (Fig. 5g, *P* < 0.001).

### Plasma concentrations of cytokines of MRL/lpr and Balb/c mice

**Fig. 6:**
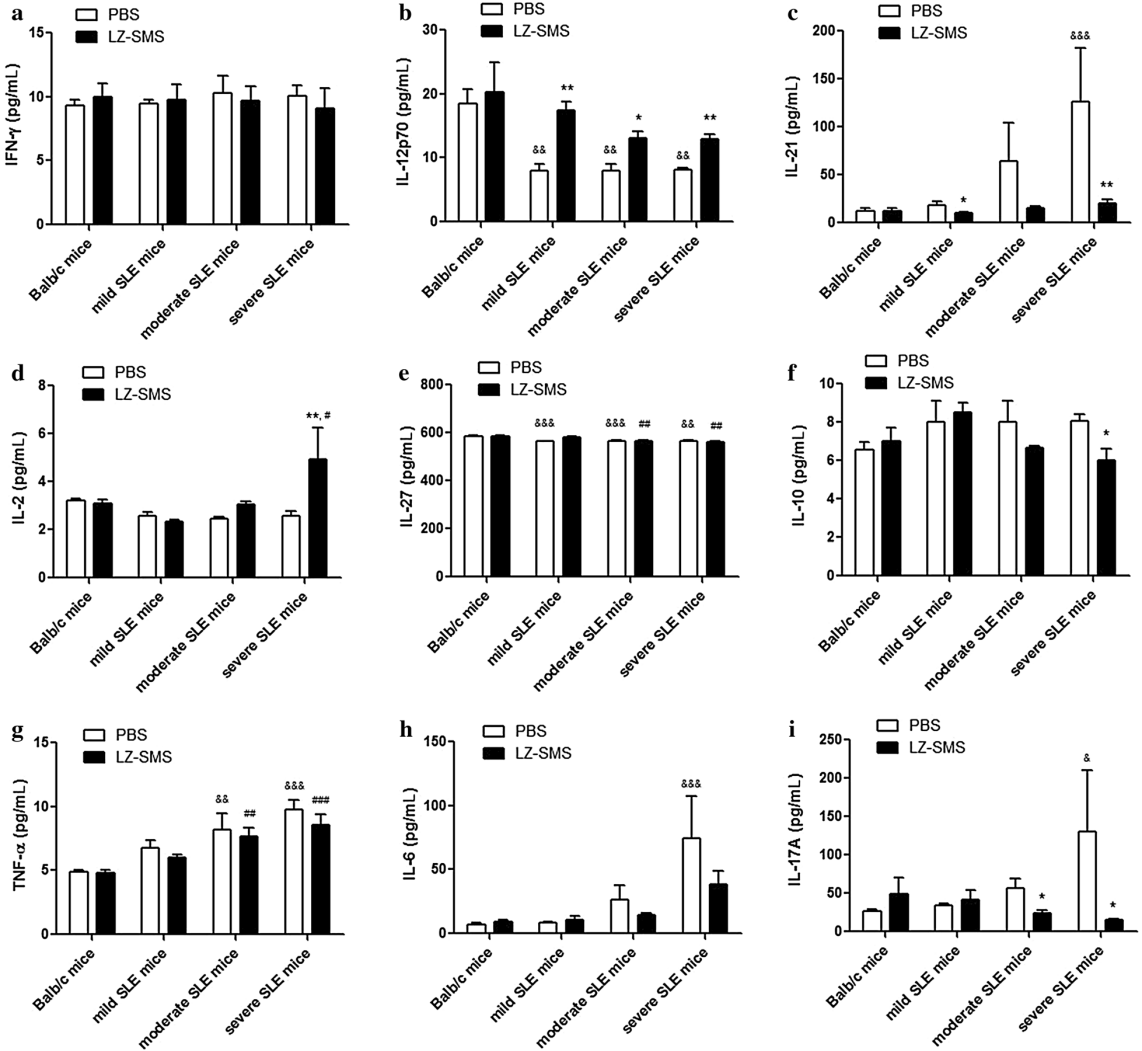
Plasma concentrations of cytokines of MRL/lpr and Balb/c mice treated with LZ–SMS or PBS. *Bar charts* show the arithmetic mean values of the plasma concentrations of **a** IFN-γ, **b** IL-12P70, **c** IL-21, **d** IL-2, **e** IL-27, **f** IL-10, **g** TNF-α, **h** IL-6 and (i) IL-17A. *P < 0.05, **P < 0.01, LZ–SMS vs. PBS treatment; ^&&^ *P* < 0.01, ^&&&^ *P* < 0.001, Balb/c control mice vs. PBS-treated MRL/lpr mice; ^#^ *P* < 0.05, ^##^ *P* < 0.01, ^###^ *P* < 0.001, Balb/c control mice vs. LZ–SMS-treated MRL/lpr mice (n = 5 in each group)

There were decreases in the plasma concentrations of the inflammatory cytokines (IFN-γ, TNF-α and IL-6) observed in the moderate and severe SLE mice treated with LZ–SMS compared with the PBS-treated mice (Fig. 6). In particular, the plasma concentrations of the Th17 cytokines IL-21 and IL-17A in the severe SLE mice decreased significantly following their treatment with the LZ–SMS material (Fig. 6c, f). In contrast, the plasma concentration of IL-2 in the severe SLE mice (*P* = 0.004), as well as the concentrations of IL-12p70 in each group of MRL/lpr mice treated with LZ–SMS increased significantly compared with those in the PBS-treated mice (Fig. 6b, d) (all *P* < 0.05). Furthermore, the plasma concentration of IL-10 in the severe SLE mice treated with LZ–SMS decreased significantly compared with that in the PBS-treated mice (Fig. 6f, *P* = 0.011). There were no significant differences in the plasma concentrations of IL-27 among the different groups of MRL/lpr mice treated with LZ–SMS compared with those in the PBS-treated mice (Fig. 6e, *P* = 0.156), even though the plasma concentrations in the moderate and severe SLE mice were significantly lower than those of the Balb/c mice (all *P* < 0.01). However, the significant differences (*P* < 0.01) observed in the IL-27 concentration in this case may not be physiologically or immunologically relevant.

## Discussion

In the present study, we have evaluated the anti-inflammatory efficacy of LZ–SMS following its oral and intraperitoneal administration in MRL/lpr mice. This LZ–SMS formulation can be suspended in PBS administered at a maximum concentration of 500 mg/kg/day [5]. MRL/lpr mice were orally administered this material on a daily basis for 7 days, followed by intraperitoneal administration for a further 7 days. Notably, none of the LZ–SMS components administered in this study exceeded the toxic doses reported in humans and mice [28, 31]. MRL/lpr mice treated with LZ–SMS showed a marked decrease in the extent of the symptoms associated with their SLE disease, including improvements in proteinuria, leukocyturia, interstitial nephritis and vessel infiltration. The eldest SLE mice (20–24 weeks) showed the most severe symptoms of lupus nephritis prior to treatment. Increases were also observed in the nephritis-related clinical and biochemical indexes of the SLE mice, along with increases in age among the three different groups (mild, moderate and severe).

No data were obtained in the present study pertaining to the absorption or systemic distribution of the active ingredients of LZ–SMS. LZ contains β-glucan, which is an immunomodulating polysaccharide capable of potential interactions with pattern recognition receptors such as dectin-1. It is noteworthy that dectin-1 is a c-type lectin, which can collaborate with TLR2 to trigger innate immune responses in antigen-presenting cells [32, 33], as well as inducing a balance between pro- and anti-inflammatory responses [34–37]. Furthermore, TLR2 signaling could potentially enhance the proliferation of Treg cells both in vitro and in vivo, which would result in the transient suppression of Foxp3 expression and the loss of their suppressive functions. It has been shown that the expression and function of dectin-1 are defective in the peripheral blood monocytes collected from patients with SLE and RA compared with healthy controls, suggesting that dectin-1 could be involved in the pathogenesis of autoimmune inflammatory conditions [38]. Dectin-1 signaling could also make a significant contribution to the expansion of Treg cells and play a critical role in the prevention of several inflammatory conditions, such as type 1 diabetes [39]. The protocol used for the intraperitoneal administration of this combination therapy was the same as that used for its oral administration, except that the dosage used in the former of these two cases was reduced to 50 mg/kg/day because of the limited solubility of LZ–SMS.

The pronounced reduction observed in the nephritis symptoms of the mice in the present study following their treatment with LZ–SMS could be attributed to the ability of LZ–SMS to down-regulate the expression of multiple inflammatory-related genes (e.g., Th17), and Th1-cell regulated genes, as well as genes associated with the expression of inflammatory cytokines and receptors, chemokines, adhesion molecules and chronic inflammation-activated proteins [40]. These responses could also be related to the ability of LZ–SMS to up-regulate the expression of regulatory iTreg-related genes and their downstream transcription factors, such as Foxp3, Stat1 and Stat4. Although the mechanism of action for the anti-nephritic efficacy of this LZ–SMS formulation remains unclear, *Lingzhi* has been reported to regulate the production of several pro-inflammatory cytokines associated with the pathogenesis of SLE in peripheral mononuclear cells [5, 15–17].

The LZ–SMS formulation used in the present study led to an increase in the percentages of immunomodulatory CD4^+^CD25^+^Foxp3^+^ Treg cells and IL-10^+^ Breg cells in the SLE mice compared with those in the PBS-treated SLE mice. These elevated levels of Treg cells could subsequently lead to (1) an increase in the frequency ratio of Treg to Teff cells; (2) an increase in the plasma concentration of IL-2, which could lead to an increase in the differentiation of Treg cells from naive T cells, the development of Treg cells in the thymus and the immunoregulatory roles of the Treg cells [41, 42]; or (3) considerable reductions in the amounts of several proinflammatory cytokines, including IL-21, TNF-α, IL-6 and IL-17A. Although the frequency and phenotypic characteristics of the Treg cells in the MRL/lpr mice were similar to those found in the normal mice, the inhibitory functions of the Treg cells in the MRL/lpr mice could be impaired by the LZ–SMS treatment [43]. Further functional assays are therefore required to develop a better understanding of the roles played by TLR2 and dectin-1 in Treg cells during the treatment of SLE in MRL/lpr mice with LZ–SMS. The overall results showed decreases in the plasma concentrations of ANA and anti-dsDNA antibody in the LZ–SMS-treated SLE mice (Fig. 3), indicating that the suppressive activity of LZ–SMS would be not only limited to CD4^+^ Treg cells, but also related to the propagation of IL-10^+^ Breg cells. However, the low frequency of IL-10^+^ Breg cells in peripheral blood cells, and the reduced plasma concentration of IL-10 in the LZ–SMS-treated severe SLE mice could limit the immunoregulatory role of LZ–SMS in this context because of the exacerbation of the SLE disease.

## Conclusion

Upon LZ–SMS treatment, the percentages of CD4^+^CD25^+^Foxp3^+^ Treg and IL-10^+^ Breg cells increased significantly in the SLE mice compared with those in the untreated mice. Furthermore, there was a decrease in the plasma concentrations of specific inflammatory cytokines, as well as a down-regulation in the expression of the corresponding cytokine-related genes in the LZ–SMS-treated mice. The clinical characteristics of the LZ–SMS-treated SLE mice were also improved significantly.

## Additional file

10.1186/s13020-016-0093-x Ethical approval document of animal experiments.

## Abbreviations

SLE: systemic lupus erythematosus
CM: Chinese medicines
LZ–SMS: *Lingzhi* and *San*-*Miao*-*San*
RA: rheumatic arthritis
FLS: fibroblast-like synoviocytes
Tregs: regulatory T cells
Bregs: regulatory B cells
ANA: anti-nuclear antibody
anti-ds-DNA: anti-double stranded DNA antibody
PAS: periodic acid-Schiff
ELISA: enzyme-linked immunosorbent assay
qPCR: quantitative real-time polymerase chain reaction
TNF-α: tumor necrosis factor-α
IFN-γ: interferon-γ
SD: standard deviation
IQR: interquartile range
