# Supplementary material for: Anti-inflammatory activities of Ganoderma lucidum (Lingzhi) and San-Miao-San supplements in MRL/lpr mice for the treatment of systemic lupus erythematosus
Source: Chin Med. 2016 Apr 29;11:23. doi: 10.1186/s13020-016-0093-x (PMC4851790; doi:10.1186/s13020-016-0093-x)
Supplement: Supplementary file 1 — 10.1186/s13020-016-0093-x Ethical approval document of animal experiments. [file 13020_2016_93_MOESM1_ESM.pdf]

Supplementary File

MS: 8847245531604429
Anti-inflammatory activities of Lingzhi and San Miao San supplementation in MRL/lpr mice: potential traditional Chinese medicine for the treatment of systemic lupus erythematosus

Zhe Cai, Chun Kwok Wong, Jie Dong, Delong Jiao, Man Chu, Ping Chung Leung, Clara Bik San Lau, Ching Po Lau, Lai Shan Tam and Christopher Wai Kei Lam

(1) Scanned copy of the ethical approval document of animal experiments

FACULTY OF MEDICINE
THE CHINESE UNIVERSITY OF HONG KONG
香港中文大學
醫 學 院

Tel 電話 : (852) 3943 6798
Fax 傳真 : (852) 2603 5011
E-mail 電郵 : aeec@med.cuhk.edu.hk
Homepage 網址 : http://www.aeec.cuhk.edu.hk

April 23, 2014

Professor WONG Chun Kwok
Department of Chemical Pathology
Faculty of Medicine

Dear Prof Wong,

Animal Experimentation Ethics Approval

I am pleased to confirm that the Animal Experimentation Ethics Committee agrees to grant ethical approval for your research project entitled "Immunoregulatory Roles of the Novel Anti-inflammatory Cytokine Interleukin-35 in Systemic Lupus Erythematosus Mice" (Ref No. 14-012-MIS).

Please also note that the approval is for the procedures described in your application and variation from these procedures requires an additional endorsement from the AEEC.

Thank you for your attention.

Yours sincerely,

Doctor ROWLANDS Dewi Kenneth
On Behalf Of Professor LAN Hui Yao
Chairman
Animal Experimentation Ethics Committee

Cc Acting Director, Laboratory Animal Services Centre

Serving the community through quality education, caring practice, and advancement of health sciences

(2) Protocol that was submitted for the ethical approval of the animal studies

Inbred female SLE mice (MRL/MpJ-Faslpr/2J) (n = 120, 12-24 weeks old) will be fed with normal chow diet and maintained in a temperature-controlled room with a 12-hour light/dark cycle. Peritoneal cells, spleen, kidneys, thymus and serum will be harvested as control from 20-24 weeks old normal Balb/c mice (n = 40). Briefly, MRL/MpJ-Faslpr/2J mice will begin to develop the clinical sign of lupus on day 1. On day 4, serum harvested from each group of mice using retro-orbital blood collection will be kept frozen at -70℃ for later tests of anti-nuclear antigen antibody (ANA), cytokines and chemokines. Urinary protein will also be recorded daily. SLE and normal control mice will be given 10 daily intravenous injections of 2 µg/200 µl of murine recombinant IL-35 or 10 times of 200µl PBS from day 8- day 17. Mice will be killed one day (day 18) by cervical dislocation and serum will be obtained by cardiac puncture for the serum assay of biochemical parameters including ANA, anti-double stranded DNA antibody, anti-single stranded DNA antibody, complement C3/C4, immunoglobulin, cytokines and chemokines. Results will then be compared between SLE and normal control BALB/c mice (n = 20 each treatment group).

(3) The information (including approval procedures and membership) about the Animal Experimentation Ethics Committee of The Chinese University of Hong Kong that complying with international guidelines.

For the above information, please browse the below website of University Animal Experimentation Ethics Committee (AEEC), The Chinese University of Hong Kong.

http://www.aeec.med.cuhk.edu.hk/member.html
